# Race and Neighborhood Access to Retailers Offering SNAP Incentives for Produce

**DOI:** 10.1001/jamanetworkopen.2026.4218

**Published:** 2026-04-01

**Authors:** Jared Grant, Joel Cuffey

**Affiliations:** 1 The Ohio State University, Columbus; 2 Auburn University, Auburn, Alabama

## Abstract

This cross-sectional study evaluates whether there is an association between the racial composition of a neighborhood and its access to produce incentives for Supplemental Nutrition Assistance Program (SNAP) recipients.

## Introduction

The Gus Schumacher Nutrition Incentive Program (GusNIP) is the primary federal program seeking to improve diets among households participating in the Supplemental Nutrition Assistance Program (SNAP) by incentivizing the purchase of fruits and vegetables at specific retailers.^1,2^ Communities with predominantly Black residents have historically experienced a lack of resources and structural disinvestment, which may limit access to retailers participating in GusNIP.^1^ We investigated whether access to retailers participating in GusNIP is associated with neighborhood racial composition.

## Methods

This cross-sectional study used secondary data and followed the STROBE reporting guideline. It was determined exempt from ethics review by the Ohio State University’s institutional review board. We obtained addresses of retailers offering GusNIP incentives between September 2019 and September 2023 from the GusNIP Nutrition Incentive Program Training, Technical Assistance, Evaluation, and Information Center. GusNIP retailer formats could be farm direct, such as farmers markets, or brick and mortar, such as grocery stores. We geocoded these addresses and measured the straight-line distance between the population-weighted centroid of each 2010 census tract and the closest GusNIP retailer of any format. We created binary indicators for whether a tract’s centroid was within 0.5, 1.0, 5.0, and 10.0 miles of a GusNIP retailer.

Estimates of tract sociodemographics were from the 2015 to 2019 American Community Survey, which collects information on self-identified race and ethnicity.^3^ Using the estimated percentage of each tract’s population identifying as non-Hispanic Black (hereafter, Black), we created indicators for whether the tract’s percentage of Black residents was in the first, second, third, or fourth quartile.

We used 4 linear probability models to describe adjusted differences in access to GusNIP stores. Outcomes were indicators for whether the tract had a GusNIP store within 0.5, 1.0, 5.0, or 10.0 miles. The independent variables of interest were indicators for quartiles 2, 3, and 4 of Black percentage. Standard errors were clustered at the county. We reported the 95% CIs and *P* values. Results were considered significant at a 2-sided *P* ≤ .05. Data were analyzed in R version 12.1 + 563 (R Project for Statistical Computing) and ArcGIS Pro version 3.3.0 (Esri).

## Results

We included 73 669 census tracts; 9403 tracts (12.8%) were within 1.0 mile of a GusNIP retailer, and 45 706 tracts (62.0%) were within 10.0 miles (Table). Tracts in the highest quartile of percentage of Black residents were less likely to be within 0.5 miles (marginal effect [ME], −0.010; 95% CI, −0.014 to −0.006; *P* < .001) and 1.0 mile (ME, −0.023; 95% CI, −0.029 to −0.016; *P* < .001) of a GusNIP store relative to tracts in the first quartile, but more likely to be within 5.0 miles (ME, 0.042; 95% CI, 0.031 to 0.052; *P* < .001) and 10.0 miles (ME, 0.068; 95% CI, 0.057 to 0.079; *P* < .001) (Figure). Compared with tracts in the first quartile of percentage of Black residents, tracts in the third quartile were less likely to be within 1.0 mile (ME, −0.014; 95% CI, −0.020 to −0.008; *P* < .001) of a GusNIP retailer, but more likely to be within 5.0 miles (ME, 0.015; 95% CI, 0.005 to 0.024; *P* = .02) and 10.0 miles (ME, 0.032; 95% CI, 0.022 to 0.041; *P* < .001).

**Table.** Characteristics of Census Tracts by Distance to GusNIP Retailers

Variable	Mean (SD)				
	All tracts (N = 73 669)	Tract distance to closest GusNIP retailer			
		Within 0.5 miles (n = 3615)	Within 1.0 mile (n = 9403)	Within 5.0 miles (n = 33 415)	Within 10.0 miles (n = 45 706)
Distance to nearest GusNIP retailer, miles	31.7 (130.6)	0.32 (0.12)	0.58 (0.25)	2.03 (1.33)	3.41 (2.66)
Black residents by quartile, %					
1	25.0 (43.3)	10.8 (31.0)	12.6 (33.2)	15.4 (36.1)	17.9 (38.3)
2	25.0 (43.3)	23.5 (42.4)	24.2 (42.8)	26.4 (44.1)	26.9 (44.3)
3	25.0 (43.3)	24.3 (42.9)	25.4 (43.6)	27.5 (44.6)	27.4 (44.6)
4	25.0 (43.3)	41.4 (49.3)	37.8 (48.5)	30.7 (46.1)	27.8 (44.8)
Non-Hispanic White residents, %	57.3 (29.7)	39.1 (29.3)	41.5 (29.4)	48.2 (29.4)	51.8 (29.5)
Hispanic residents, %	17.2 (21.0)	24.0 (24.1)	22.9 (23.6)	21.1 (22.7)	20.2 (22.4)
Residents belonging to additional non-Hispanic groups, %^a^	10.6 (11.2)	14.6 (14.7)	14.7 (14.7)	13.4 (13.1)	12.5 (12.1)
Household owns car, %	88.8 (17.7)	67.7 (25.4)	75.7 (22.6)	85.9 (17.7)	88.2 (16.3)
Uses public transportation, %	5.2 (11.5)	25.8 (25.5)	18.9 (22.4)	9.6 (15.6)	7.7 (13.9)
Households with annual income <poverty limit, %	14.3 (11.6)	21.7 (13.9)	20.2 (13.8)	16.1 (12.8)	14.7 (12.1)
Households in rural tract, %	36.7 (48.2)	6.8 (25.1)	8.3 (27.7)	15.9 (36.6)	22.0 (41.4)
Education, %					
Less than high school	12.4 (10.4)	16.9 (13.3)	15.9 (12.6)	13.4 (11.4)	12.6 (11.0)
High school	27.3 (11.7)	23.9 (11.8)	24.5 (11.7)	25.0 (11.2)	25.3 (11.0)
Some college	28.3 (8.9)	24.0 (9.7)	25.4 (9.50)	27.5 (9.1)	28.0 (8.80)
College	18.5 (10.6)	20.1 (12.1)	19.9 (11.8)	20.1 (11.1)	20.2 (10.8)
Greater than college	11.6 (10.2)	14.4 (13.4)	13.7 (12.7)	13.3 (11.6)	13.2 (11.1)
Not in labor force, %	36.6 (11.4)	35.5 (11.6)	35.7 (11.1)	35.7 (10.4)	35.7 (10.2)
Unemployed, %	5.7 (4.4)	7.4 (5.7)	7.2 (5.7)	6.3 (4.9)	5.9 (4.5)
Gini Index	41.7 (8.8)	46.1 (8.2)	44.9 (8.1)	42.8 (7.9)	42.2 (7.7)
Population density, person/km^2^	2.1 (4.73)	10.5 (13.3)	7.34 (10.6)	3.70 (6.55)	3.03 (5.76)

Abbreviation: GusNIP, Gus Schumacher Nutrition Incentive Program.

^a^ Included American Indian or Alaska Native, Asian, Native Hawaiian or Other Pacific Islander, and other racial groups.

**Figure.**
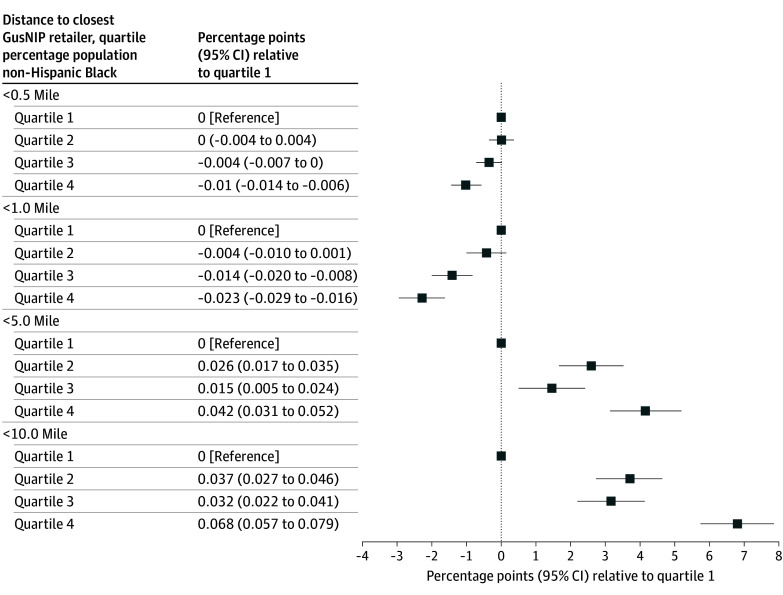
Adjusted Association Between Distance to a Gus Schumacher Nutrition Incentive (GusNIP)–Participating Retailer and Tract Racial Composition Among 73 669 Tracts Adjusted associations from linear probability models of indicators for presence of a GusNIP retailer within successive distances on indicators for Black percentage quartile. Models controlled for racial composition (Hispanic, non-Hispanic American Indian or Alaska Native, non-Hispanic Asian, non-Hispanic Native Hawaiian or Other Pacific Islander, or other non-Hispanic race), percentage of households owning a car, percentage of employed population taking public transportation to work, percentage of population not in the labor force, poverty rate, income Gini index, unemployment rate, population density, and an indicator for whether the tract has any rural population. Control variables were restricted to those with variance inflation factors less than 10.

## Discussion

This study found that GusNIP retailers were not located in close proximity to communities with a higher proportion of Black residents but were located in broader areas where Black individuals live. Study limitations include the use of straight-line instead of travel-time distance measures, the potential for unobserved confounders, and access to potentially different GusNIP retail formats across communities. Results suggest practices such as historical redlining^4^ or restrictive zoning^5^ may have resulted in fewer stores with the ability to participate in GusNIP being located in communities with higher proportions of Black residents or reduced the ability of these communities to organize, find match funding, or administer GusNIP projects.

## Notes

Supplement.
Data Sharing Statement

## References

[1] John S, Melendrez B, Leng K, Nelms A, Seligman H, Krieger J. Advancing equity in the Farm Bill: opportunities for the Gus Schumacher Nutrition Incentive Program (GusNIP). Nutrients. 2023;15(23):4863. doi:10.3390/nu15234863 38068722 PMC10707921

[2] Moran A, Thorndike A, Franckle R, . Financial incentives increase purchases of fruit and vegetables among lower-income households with children. Health Aff (Millwood). 2019;38(9):1557-1566. doi:10.1377/hlthaff.2018.05420 31479362

[3] Manson S, Schroeder J, Van Riper D, . IPUMS National Historical Geographic Information System: Version 19.0 [dataset]. 2024. Accessed February 18, 2026. https://www.ipums.org/projects/ipums-nhgis/d050.V19.0

[4] Yang Y, Cho A, Nguyen Q, Nsoesie EO. Association of neighborhood racial and ethnic composition and historical redlining with built environment indicators derived from Street View images in the US. JAMA Netw Open. 2023;6(1):e2251201. doi:10.1001/jamanetworkopen.2022.51201 36652250 PMC9856713

[5] Shertzer A, Twinam T, Walsh RP. Zoning and segregation in urban economic history. Reg Sci Urban Econ. 2022;94:103652. doi:10.1016/j.regsciurbeco.2021.103652

