# Supplementary material for: Race and Neighborhood Access to Retailers Offering SNAP Incentives for Produce
Source: JAMA Netw Open. 2026 Apr 1;9(4):e264218. doi: 10.1001/jamanetworkopen.2026.4218 (PMC13044651; doi:10.1001/jamanetworkopen.2026.4218)
Supplement: Supplement. — Data Sharing Statement [file jamanetwopen-e264218-s001.pdf]

Data Sharing Statement

Grant. Race and Neighborhood Access to Retailers Offering SNAP Incentives for Produce. *JAMA Netw Open*. Published April 01, 2026. doi:10.1001/jamanetworkopen.2026.4218

Data
Data available: Yes
Data types: Data (not involving human participants), Data dictionary
How to access data: Request for data can be sent to Joel Cuffey (cuffey@auburn.edu).
When available: With publication

Supporting Documents
Document types: Statistical/analytic code
How to access documents: Request for code can be sent to Joel Cuffey (cuffey@auburn.edu).
When available: With publication

Additional Information
Who can access the data: Researchers with uses of the data that have been approved by this study's research team may ask for data/code.
Types of analyses: Any purpose
Mechanisms of data availability: Data/Code will be made available by email without additional support from the study team.
